# Pseudoaneurysm of Lumbar Artery following a Vertebral Biopsy: A Case Report

**DOI:** 10.1155/2012/127124

**Published:** 2012-01-26

**Authors:** Yutaka Mifune, Masayoshi Yagi, Yasunobu Iwasaki, Minoru Doita

**Affiliations:** ^1^Department of Orthopaedic Surgery, Shin-Suma Hospital, Kobe 654-0047, Japan; ^2^Department of Orthopaedic Surgery, Kobe University Graduate School of Medicine, 5-7-2 Kusunoki-cho, Chuo-ku, Kobe 650-0047, Japan

## Abstract

A 74-year-old man developed a severe low back pain and a fever. In the initial examinations, a collapse of the L5 anterosuperior vertebral body and narrowing of the L4/5 disc space were identified on radiographs, and the laboratory data showed inflammatory results. A computed tomography (CT) and a magnetic resonance imaging showed collapse of L5. A needle biopsy was performed to make a diagnosis; however, an abdominal pain and a hypotension appeared after the biopsy. An abdominal CT showed a hematoma in the retroperitoneal space, and an angiography revealed a left fourth lumbar artery pseudoaneurysm. The pseudoaneurysm was treated with transcatheter placement of microcoils. Although haemorrhagic complications following needle biopsy are very rare, patients with large amounts of vertebral destruction may have unusual anatomical positions of the lumber artery. Therefore, surgeons should be aware of the possibility of lumbar artery injury during a needle biopsy and take care of prebiopsy plans.

## 1. Introduction

Percutaneous needle biopsy of vertebral bodies through posterior approach, relatively simple and safe technique with low complication rate, is widely used in cases of suspected spondylitis or tumor metastasis of spine [1]. However, some problems remain with such needle biopsies, one of which is neural or vascular damage. Haemorrhagic complication of lumbar arteries following needle biopsy is uncommon, and only few cases have been reported [2, 3]. We describe an unusual case of 74-year-old man who developed pseudoaneurysm of fourth lumbar artery following vertebral body needle biopsy.

## 2. Case Report

**Figure 1:**
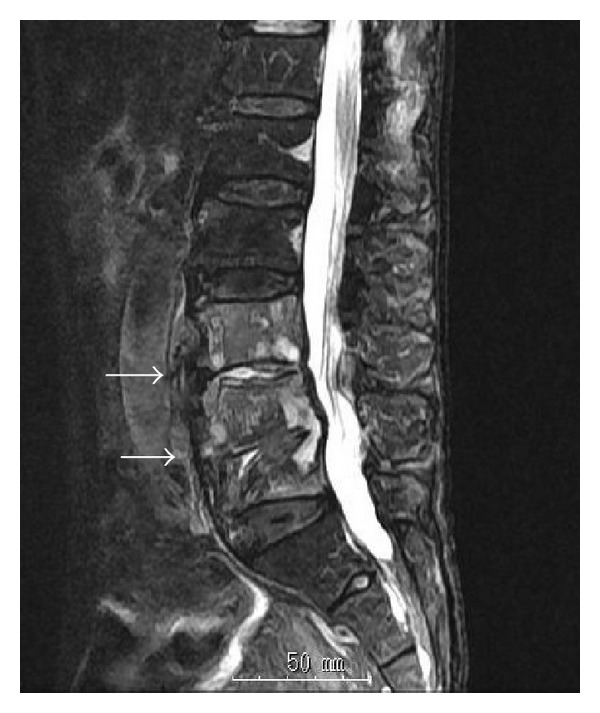
MRI showed the collapse of L5 and an increased signal intensity in the L3, 4, 5 vertebral body on T2 STIR images (arrow).

A 74-year-old man suffering from low back pain for 6 months visited another hospital. His condition worsened, and a week before being admitted to our institution, he gradually developed severe low back pain for no particular reason, accompanied by fever (38.5°C). Antibiotics (cefazolin sodium, 2 g/day intravenously) were prescribed at the previous hospital, and he was transferred to our clinic. Due to severe back pain, the patient could not sit upright. No neurological abnormalities were found, and laboratory data showed high white blood cell count (13800/*μ*L), high C-reactive protein (19.35 mg/dL), and low hemoglobin levels (10.8 g/dL). On the lumbar radiograph, collapse of the L5, anterosuperior vertebral body and narrowing of the L4/5 disc space were identified. Computed tomography (CT) showed the collapse of L5, and magnetic resonance imaging (MRI) further demonstrated decreased signal intensity in the L3, 4, 5 vertebral bodies on T1-weighted images, intermediate signal intensity on T2-weighted images, and increased signal intensity on T2 STIR images (Figure 1).

For the purpose of a diagnosis, vertebral biopsy from the L4, 5 body and L4/5 disc was performed using a threaded bone biopsy trocar (Ostycut, Angiomed) through posterolateral approach under local anesthesia. The patient was positioned in the right lateral decubitus position. All procedures were performed under the biplane fluoroscopic control, and there were no immediate complications. The histology showed nonspecific reactive hyperplasia with no malignancy, and the culture tests were all negative.

**Figure 2:**
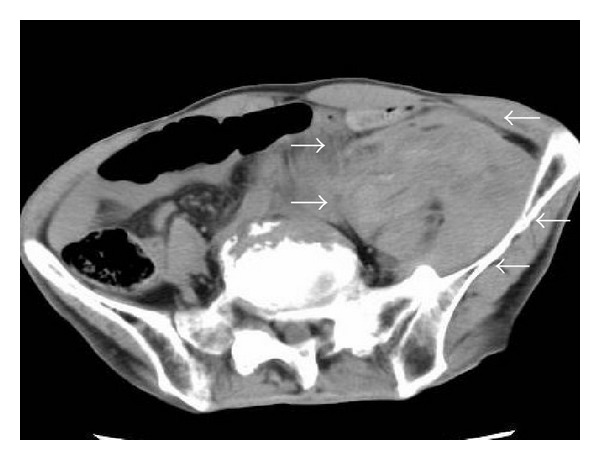
An abdominal CT showed about a 6 cm hematoma in the retroperitoneal space (arrow).

**Figure 3:**
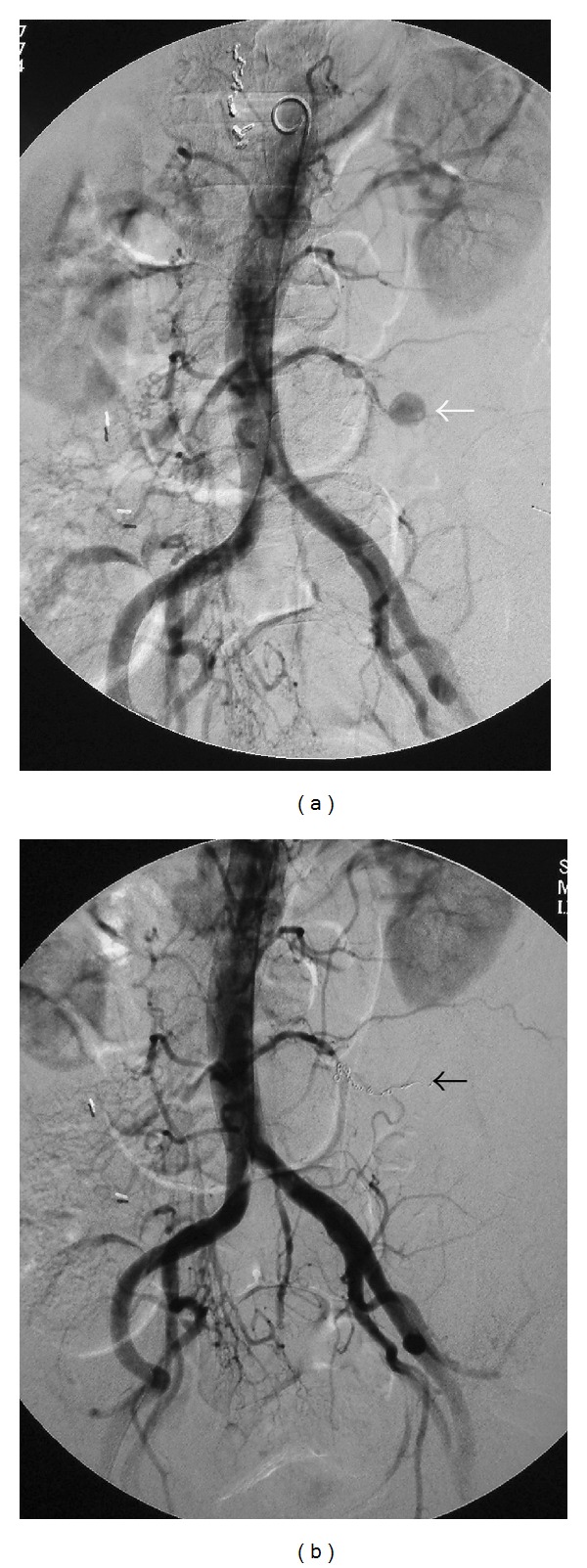
(a) Angiogram revealed a pseudoaneurysm of left fourth lumbar artery (white arrow). (b) The pseudoaneurysm was treated with transcatheter placement of microcoils into the left fourth lumbar artery (black arrow).

However, the next day of the biopsy, the patient experienced a left lower abdominal pain and a hypotension, and hemoglobin levels decreased to 7.0 g/dL. An abdominal CT showed approximately 6 cm hematoma in left retroperitoneal space (Figure 2). Moreover, angiography revealed left fourth lumbar artery pseudoaneurysm (Figure 3(a)). The pseudoaneurysm was treated with transcatheter placement of microcoils (VORTEX, GDC, Boston-Scientific Japan) into left fourth lumbar artery (Figure 3(b)). After coil embolization, the patient's abdominal pain decreased. After the treatment for the pseudoaneurysm, the patient was treated with a rest and antibiotics for a month, leading to both subjective improvement and fall in white blood cell and C-reactive protein.

## 3. Discussion

Lumbar needle biopsy is generally regarded as safe technique with low complication rate reported between 0 and 4% including vascular injury, soft tissue recurrence of tumor, and infection [1, 2]. Haemorrhagic complications following biopsy are reported to be very rare, and lumbar artery injury is extremely rare [1]. To our knowledge, only one case in which a pseudoaneurysm has been produced as a result of vertebral biopsy has been described in the English literature [3].

**Figure 4:**
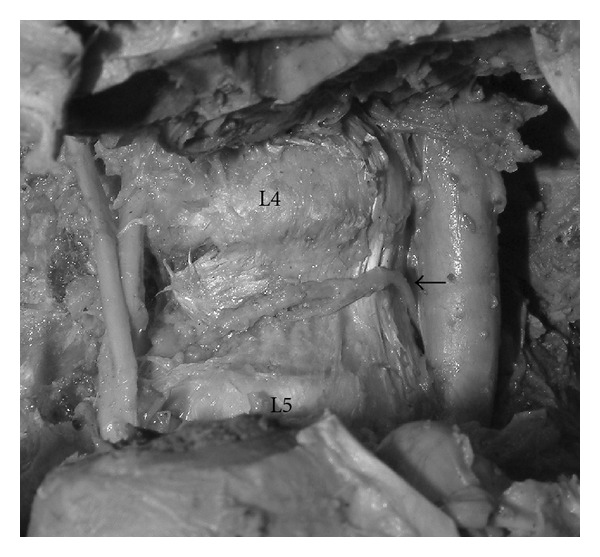
The lumbar arteries of L1 to L4 are small paired vessels that originate from the dorsal aspect of the abdominal aorta at the level of the transverse processes (arrow). These vessels run laterally along the bodies of the lumbar vertebrae.

The lumbar arteries of L1 to L4 are small paired vessels that originate from the dorsal aspect of the abdominal aorta at the level of the transverse processes [3] (Figure 4). The lower lumbar arteries occasionally originate from common trunk near the midline of posterior aorta. These vessels run laterally along the bodies of the lumbar vertebrae and divide into anterior and posterior branches at the medial border of the psoas muscle [3]. From an anatomical view point, it would be less possible that a penetration at the level of disc or inferior vertebrae edge cannot incur a lumbar artery injury. However, a patient with a large amount of vertebral destruction may have an unusual anatomical position. As shown in the present case, it may be difficult for surgeons to comprehend the location of the lumbar artery during a lumbar biopsy. Many case reports of spinal biopsy under CT guidance indicate its efficacy for reducing complications and collecting foci more accurately [4, 5]. However, it has been also pointed out that the difficulty in nerve root and segmental artery visualization with CT leads to the risk of nerve or vascular injuries. Ashizawa et al. used a conventional C-arm image intensifier to guide a thin trocar into the medullary space of the pedicles and into the vertebral involvements [2]. This turned out to be not so difficult when the procedure was checked both on A-P and lateral view. Therefore, a safe route for the biopsy needle into the vertebral body without neural or vascular damage should be considered.

An endovascular embolization has been described as the most appropriate treatment for lumbar artery pseudoaneurysms as it avoids the risks associated with another anesthesia, surgical incision, and the difficulty of locating and controlling the bleeding [3, 6, 7]. A recent paper indicated that successful selective embolization of abnormal vessels was performed in eleven patients suffering from a symptomatic lumbar [8]. In our case, a transcatheter embolization also successfully eradicated the pseudoaneurysm.

To make a rapid diagnosis of haemorrhagic complications following biopsy, it is important to check the clinical symptoms and vital signs of the patient carefully after procedures. In patients with suspected vascular injury, multiphasic contrast-enhanced CT is obligatory for diagnosing retroperitoneal bleeding. A homogenous high-density mass on CT imaging is suggestive of blood collection. If a retroperitoneal hematoma is suspected, angiography can be used to detect active bleeding of an artery in a seemingly stable patient [3, 9, 10]. In the present case, a left lower abdominal pain and a hypotension appeared a day after biopsy, and both abdominal CT and angiography were immediately performed and proved to be very effective for accurate diagnosis and prompt treatment.

Recently, a few reports have described that percutaneous suction aspiration with drainage is effective for pyogenic spondylitis [11]. In such cases, surgeons also should note the possibility of lumbar artery injury. Biafora et al. said that injury to lumbar artery during percutaneous procedures may be difficult to prevent [12]. Such possible complications should be carefully explained to the patient prior to the operation. A careful prebiopsy plan is required to avoid complications. Additionally, increased awareness of this complication will decrease associated morbidity.
